# Use of the proteomic tool MALDI-TOF MS in termite identification

**DOI:** 10.1038/s41598-021-04574-0

**Published:** 2022-01-14

**Authors:** Bouthaina Hasnaoui, Adama Zan Diarra, Jean-Michel Berenger, Hacène Medkour, Ahmed Benakhla, Oleg Mediannikov, Philippe Parola

**Affiliations:** 1 Aix Marseille Univ, IRD, SSA, AP-HM, VITROME, Marseille, France; 2 IHU-Méditerranée Infection, Marseille, France; 3 Aix Marseille Univ, IRD, AP-HM, MEPHI, Marseille, France; 4 Département des Sciences Vétérinaire, Université Chadli Bendjdid, 36000 El Tarf, Algeria

**Keywords:** Biochemistry, Molecular biology, Zoology

## Abstract

Matrix-assisted laser desorption/ionization mass spectrometry (MALDI-TOF MS) has proved effective for the identification of many arthropods. A total of 432 termite specimens were collected in Mali, Cote d’Ivoire, Togo, Senegal, Switzerland and France. Morphologically, 22 species were identified, including *Ancistrotermes cavithorax, Amitermes evuncifer*, *Cryptotermes brevis*, *Cubitermes orthognathus*, *Kalotermes flavicollis*, *Macrotermes bellicosus*, *Macrotermes herus*, *Macrotermes ivorensis*, *Macrotermes subhyalinus*, *Microcerotermes parvus*, *Microtermes* sp., *Odontotermes latericius*, *Procubitermes sjostedti*, *Promirotermes holmgreni*, *Reticulitermes grassei*, *Reticulitermes lucifugus*, *Reticulitermes santonensis*, *Trinervitermes geminatus*, *Trinervitermes occidentali*s, *Trinervitermes togoensis*, *Trinervitermes* sp., *Trinervitermes trinervoides* and *Trinervitermes trinervius*. Analysis of MALDI-TOF MS spectra profiles from termites revealed that all were of high quality, with intra-species reproducibility and inter-species specificity. Blind testing of the spectra of 389 termites against our updated database with the spectra of 43 specimens of different termite species revealed that all were correctly identified with log score values (LSVs) ranging from 1.65 to 2.851, mean 2.290 ± 0.225, median 2.299, and 98.4% (383) had LSVs > 1.8. This study is the first on the use of MALDI-TOF for termite identification and shows its importance as a tool for arthropod taxonomy and reinforces the idea that MALDI-TOF MS is a promising tool in the field of entomology.

## Introduction

Termites or white ants belong to the arthropods that have thrived on earth for over 300 million years^1,2^. They belong to the class Insecta, infra-order Isoptera^3^. Phylogenetic studies have indicated that the nearest relatives are cockroaches, which explains their classification in the order of *Blattodea*^4^. Their distribution depends on climatic conditions, especially temperature and precipitation^5^. Termites live in colonies divided into two castes (reproductive or sexual and sterile or asexual)^6,7^. Humans and termites live in close proximity^8^. Many termite species are recognized as harmful.

Termites attack the wooden parts of buildings and construction, causing damage costing more than three billion dollars each year, as well as in the agriculture field by eating fast-growing plants^9,10^. At the same time, they are known as ecosystem engineers by influencing the distribution of natural resources, such as water and nutrients in the ecosystem^11^, and that refers to their ability to generate valuable biogenesis that improves soil properties^12^, which increases water infiltration rates^13,14^. They are also of interest in traditional medicine, particularly for suturing wounds and treating angina, fever, burns and abscesses^15^. Termite species such as *Macrotermes bellicosus* have been shown to have anti-inflammatory and analgesic effects^16^. Termites can be used as bait to catch fish and birds^15^ and as a natural human food resource that has a significant value in protein and vitamins^17^. They participate in the beneficial chemical variation of the earth and its components^18^.

Morphological identification of termites, based mainly on the observation of morphological characteristics, is limited by the need for entomological expertise; i.e., the difficulty in identification up to the species level due to the ambiguity of their features, crypto-biotic social structure and their similarity, the availability of identification keys and the long time required for identification. Identification based on molecular amplification and sequencing of genes such as mitochondrial cytochrome oxidase subunits I and II *(cox1 and cox2*), genes coding for the NADH-ubiquinone oxidoreductase chain 1 (*ND1*), *Internal Transcribed Spacer (ITS2)*, large and small ribosomal *RNA* subunits (*16S and 12S rRNA*), and nuclear DNA such as *18S*, Microsat and genes for endo-beta-1,4-glucanase (*RsEG*),interactive domain-containing protein 1A (*AT-rich DNA*); these molecular markers have proven to be an efficient alternative for species identification and overcoming morphological limitations^19^. However, the molecular identification approach is still limited by the high cost of reagents, the time consumed, and the absence of universal primers allowing for the amplification of a given gene in all species and sequences of all species on GenBank^20^.

To overcome the difficulties of morphological and molecular identification of arthropods, MALDI-TOF MS has been proposed as an alternative identification technology to these two methods. The MALDI-TOF MS is a technique that allows identifying an organism from protein signals (borrowed protein) of molecular weight between 2000 and 20,000 Da. This method has been used in many studies to identify different arthropods, including ticks, mosquitoes, biting midges, fleas, lice, bedbugs, triatomines and phlebotomine sand flies, and also for the determination of their blood meal origin and to discriminate between the infectious status of some arthropod vectors^21^. In medical entomology, the use of MALDI-TOF MS requires a development of protocols such as the choice of the compartment to be used and the quantity of crushing mix allowing to have spectra with intra-species reproducibility and inter-species specificity. The part of the arthropod to generate reproducible and specific spectra by MALDI-TOF MS analysis varies between arthropod groups but also according to the developmental stages of the arthropod^21^. For example, the legs are used for mosquitoes and ticks, the cephalothorax for fleas, lice and bedbugs^21^. MALDI-TOF is a fast and easy technique that does not require expertise in entomology. However, the high cost of the machine, its maintenance, the choice of the compartment used for the analysis and the preservation methods are the limiting factors of this technique^21^. The machines used in entomology research are those used in the microbiology platform, at no extra cost, and the cost of the analysis is very low. At present, for termites the MALDI-TOF MS tool has been used to identify methanotrophic bacteria in the gut^22^, cuticular hydrocarbons^23^, carboxy-methyl cellulose, crystalline celluloses or xylan from the gut of *Reticulitermes santonensis*^24^ and the chemical profile and antimicrobial activity of *Macrotermes bellicosus* used in traditional medicine^25^. However, no study has been done on the identification of termite species by MALDI-TOF MS. Hence, the aim of this study is to evaluate the ability of MALDI-TOF to identify different species of termites collected in four West African countries and in two countries in Europe.

## Results

### Termite collection and morphological identification

**Figure 1:**
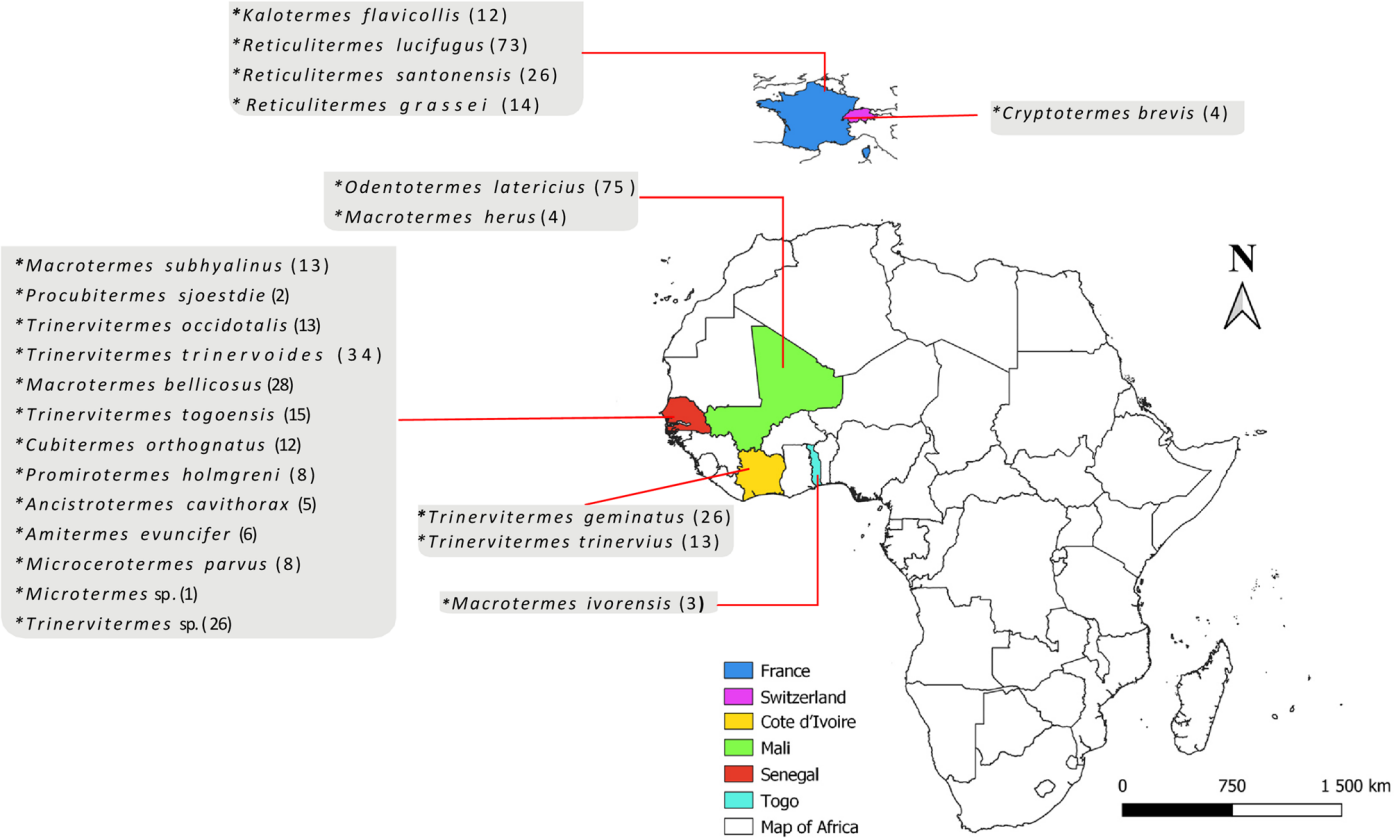
Map of Africa and part of Europe showing termite collection sites, morphologically identified termite species names and numbers of each species.

A total of 432 termite specimens were collected, including 135 in Marseille (32%), 123 in Senegal (28%), 87 in Cote d’Ivoire (20%), 79 in Mali (18%), four in Switzerland (1%) and four in Togo (1%). Morphologically, the termites were identified as belonging to 23 species, including five *Ancistrotermes cavithorax*, six *Amitermes evincifer*, 12 *Cubitermes orthognathus*, four *Cryptotermes brevis*, 12 *Kalotermes flavicollis*, 28 *Macrotermes bellicosus*, four *Macrotermes herus*, three *Macrotermes ivorensis*, 13 *Macrotermes subhyalinus*, eight *Microcerotermes parvus*, one *Microtermes* sp, 75 *Odontotermes latericius*, eight *Promirotermes holmgreni*, two *Procubitermes sjostedti,* 14 *Reticulitermes grassei*, 84 *Reticulitermes lucifugus*, 26 *Reticulitermes santonensis,* 26 *Trinervitermes geminatus*, 13 *Trinervitermes occidentalis*, 26 *Trinervitermes* sp., 15 *Trinervitermes togoensis,* 34 *Trinervitermes trinervoides* and 13 *Trinervitermes trinervius* (Fig. 1 and Table 1). The largest number of species was identified in Senegal, with 12 different species (Fig. 1 and Table 1). The termites belonged to the sterile caste (workers and soldiers) and reproductive caste (winged). We could not identify several specimens of *Trinervitermes* and *Microtermes* termites up to the species level (Table 1).

**Table 1:** Morphological and molecular identification of termites, number of each termite species, number tested by molecular and *12S rRNA* and *COI* accession number.

Morphological identification (number identified)	Number tested by molecular biology	Molecular identification			
		Closest GenBank match (% identity) 12S rRNA	Accession number	Closest GenBank match (% identity) *COI*	Accession number
*Ancistrotermes cavithorax* (5)	1	*Ancistrotermes crucifer* (98.7%)	DQ441630	*A. cavithorax* (99.3%)	AY127715
*Amitermes evuncifer* (6)	1	*A. evuncifer* (97.7%)	DQ441626	*A. evuncifer* (99.3%)	AY127718
*Cryptotermes brevis* (4)	2	*C. brevis* (100%)	KM618724	*C. brevis* (99.6%)	MT040337
*Cubitermes orthognathus* (12)	2	*Cubitermes ugandensis* (98.5%)	KP091689	*Nitiditermes* sp. (99.5%)	MN646703
*Kalotermes flavicollis* (12)	2	*K. flavicollis* (99.2%)	DQ441716	*K. flavicollis* (92.6%)	EU253842
*Macrotermes bellicosus* (28)	3	*Pseudacanthotermes militaris* (93.6%)	KY224517	*M. bellicosus* (98.9%-99.5%)	AY127702
*Macrotermes herus* (4)	1	*Macrotermes* sp. (99.7) *M. subhyalinus* (99.4%)	KY224531 DQ441726	*M. subhyalinus* (98.7%) *M. herus* (98.7%)	FJ207427 FJ207441
*Macrotermes ivorensis* (3)	1	*Macrotermes* sp. (99.4%) *M. subhyalinus* (99.2%)	KY224531 JX144937	–	
*Macrotermes subhyalinus* (13)	2	*Macrotermes* sp. (99.2) *M. subhyalinus* (99%)	KY224531 JX144937	*M. subhyalinus* (98.3%) *M. herus* (98%)	FJ207427 FJ207441
*Microcerotermes parvus* (8)	1	*Microcerotermes* sp. (96.5%)	KY224578	*Microcerotermes* sp. (92.5%)	KY224573
*Microtermes* sp. (1)	1	*Microtermes sp.* (98.7%)	KY224390	*Microtermes sp.* (99.5%)	AY127713
*Odontotermes latericius* (75)	4	*Odontotermes obesus* (97.0%)	KY224493	*O. latericius* (99.5%)	AY127714
*Procubitermes sjostedti* (2)	1	*Cubitermes ugandensis* (96.9%)	KP091689	*Nitiditermes proximatus* (99.5%)	MN646731
*Promirotermes holmgreni* (8)	1	*Promirotermes pygmaeus* (96.1%)	KY224529	*Promirotermes sp* (91.5%)	KY224554
*Reticulitermes grassei* (14)	2	*R. lucifugus* (99.7%)	NC045240	*R. lucifugus* (99.5%)	NC045240
*Reticulitermes lucifugus* (84)	6				
*Reticulitermes santonensis* (26)	2				
*Trinervitermes geminatus* (26)	2	*T. geminatus* (99.5–100%)	DQ441829	*T. geminatus* (96.8–97.2%)	JF923343
*Trinervitermes trinervoides* (34)	2				JF923317
*Trinervitermes occidentalis* (13)	3	*Cortaritermes intermedius* (96.4%)	MH574830	*T. occidentalis* (95.9–96.3%)	JF923333 JF923309
*Trinervitermes togoensis* (15)	*2*				
*Trinervitermes* sp. (26)	3				
*Trinervitermes trinervius* (13)	2	*T. geminatus* (96.1–96.7%)	DQ441829	*T. geminatus* (97.2%)	JF923343

For all the species that we identified morphologically, pictures of a body and mandibular were taken and represented in the supplementary Fig. 1.

### Validation of morphological identification by molecular tools

**Figure 2:**
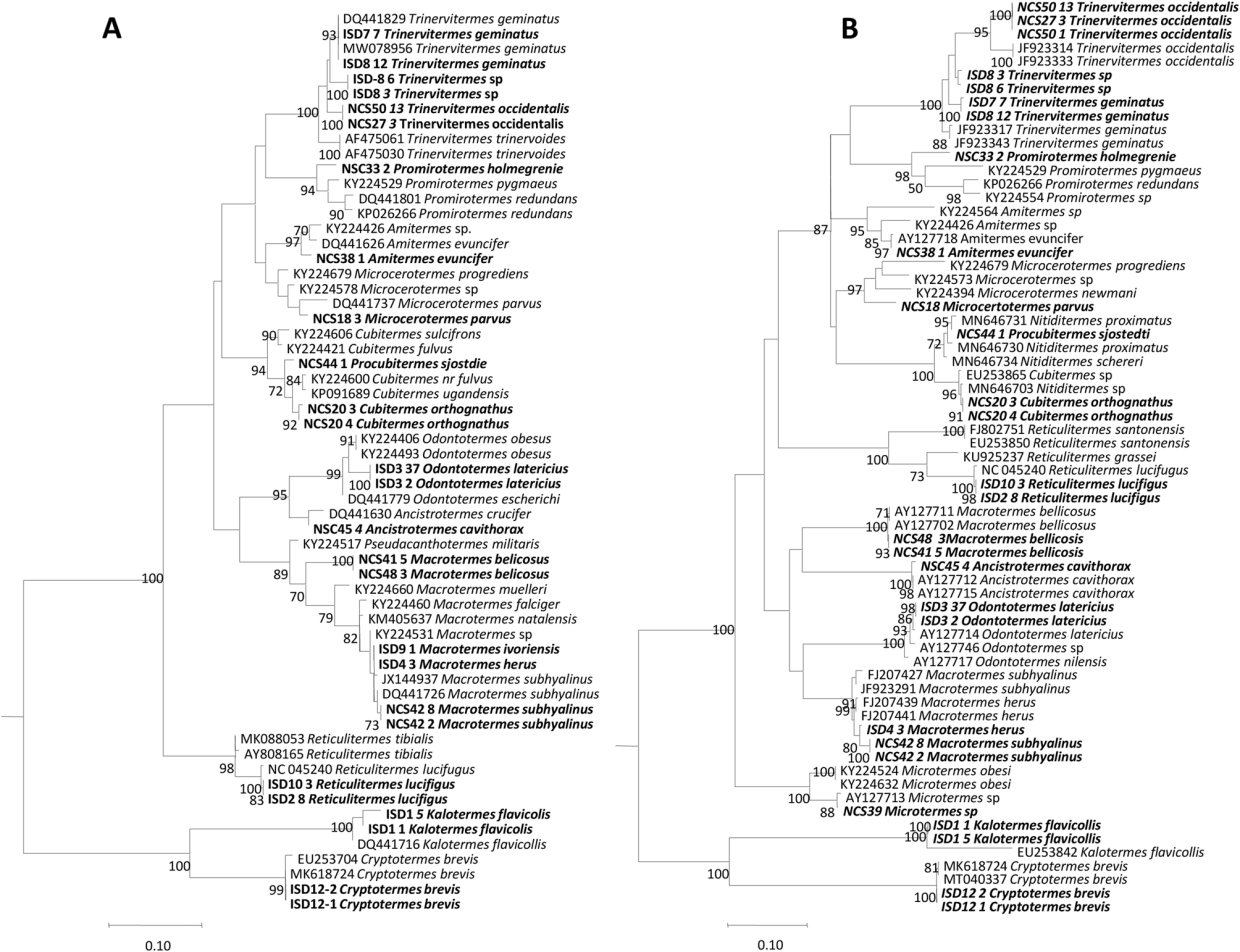
Phylogenetic tree based on *12S rRNA* gene sequences (**A**) and *COI* gene sequences (**B**). Phylogenetic tree highlighting the position of studied termites (in bold) relative to other termite sequences available via Genbank. The partial sequences of the mitochondrial *cox1* gene and the *12SrRNA* gene were aligned using CLUSTALW, and phylogenetic inferences were obtained from a maximum likelihood phylogenetic analysis with the Tamura and Nei 1993 (TrN) submodel for *12SrRNA* gene (**A**) and with the general time-reversible (GTR) submodel (**B**) proposed by TOPALi 2.5 software. The GenBank accession numbers are indicated at the beginning. The numbers at the nodes are the bootstrap values obtained by repeating the analysis 100 times to generate a majority consensus tree.

A total of 47 randomly selected termite specimens morphologically identified for the creation of our database were submitted to standard PCR and sequencing for molecular identification using both *12S rRNA* and *COI* genes. BLAST analysis of sequences obtained from specimens identified as *M. subhyalinus* showed that they were 100% and 99.4% identical to the corresponding sequence of *M. subhyalinus* (GenBank: DQ441726 and AY127708) respectively for *12S rRNA* and *COI* genes. Those identified as *M. bellicosus* were 99.7% and 93.7% identical to the corresponding sequence of *M. bellicosus* (AY127702) and *Pseudacanthotermes militaris* (KY224517). Sequences from termites identified as *M. herus*, *M. ivorensis* and *M. subhyalinus* were 99.2 to 99.7% identical to *Macrotermes* sp. (KY224531) and 99 to 99.4% to *M. subhyalinus* (DQ441726) for *12S rRNA* gene and they were also 98.3 to 98.7% identical to *M. subhyalinus* (FJ207427) and 98 to 98.7% to *M. heru*s (FJ207441) for COI gene, but we don’t obtained sequences using COI gene for *M. ivorensis*.. Specimens morphologically identified as *T. geminatus* and *T. trinervoides* were 99.5% to 100% and 97.2% identical to the corresponding sequence of *T. geminatus* (DQ441829 and JF923343), respectively for both genes. The sequences from termites identified as *T. occidentalis*, *T. togoensis* and *Trinervitermes* sp. were 96.4% and 95.9% to 96.3% identical to the corresponding sequence of *Cortaritermes intermedius* (MH574830) and *T. occidentalis* (JF923333). Sequences from termites identified as *T. trinervius* were 96.1% to 96.7% and 95.4% identical to the corresponding sequence of *T. geminatus* (DQ441829) and *T. geminatus* (JF923343), respectively for both genes. The sequences obtained from termites morphologically identified as *R. santonensis*, *R. grassei* and *R. lucifugus* were 99.7% to 99.5% identical to the corresponding sequence of *R. lucifugus* (NC_045240), respectively for both genes. The specimens identified as *K. flavicollis* were 99.2% and 91.8% identical to *K. flavicollis* (DQ441716; EU253842), respectively for both genes. The sequences from termites identified as *O. latericius* were 97% and 99.3% identical to sequence of *Odontotermes obesus* (KY224493) and *O. latericius* (AY127714), and those identified as *C. orthognathus* were identical to 99.5% to sequences of 98.4% to *Cubitermes* sp. (EU253865) and *Nitiditermes sp.* (MN646731). Termites identified as *A. evuncifer* and *Mi. parvus* were 97.7% and 99.6% identical to the corresponding sequence of *A. evuncifer* (DQ441626; AY127718) and 96.6% and 96.3% identical to *Microcerotermes* sp. *(*KY224578; F923261), respectively for both species using two genes. The sequences from termites identified as *A. cavithorax* and *P. sjostedti* were 98.7% identical to the sequence of *A. crucifer* (AY127715) and 97% to the sequence of *Cubitermes ugandensis* (KP091689), respectively for the 12S rRNA gene, 99.50% identical to the sequence of *An. cavithorax* (DQ441630), and 99.52% to the sequence of *Nitiditermes proximatus (MN646731)* for the *COI* gene. Sequences from termites identified as *Microtermes* sp. and *Trinervitermes sp* were 96.8% and 99.50% identical to the sequence of *Microtermes* sp. (KY224390 and AY127713) and 96.9% *Nasutitermes corniger* (DQ441746) and 95.9% to *T. occidentalis* (JF923309), respectively for both termite species. Those from termites identified as *P. holmgreni* were 96.9% and 91.5% identical to *Promirotermes pygmaeus* (KY224529) and *Promirotermes* sp. (KY224554) respectively for both genes. Those from *C. brevis* were 100% and 99.6% identical to *C. brevis* (KM618724 and MT040337) respectively for both genes. The results of the molecular identification of the termites in our study are summarized in Table 1. The phylogenetic position of termites in this study is shown in Fig. 2A and B. The sequences of the *12S rRNA* and *COI* genes obtained in this study were deposited in the GenBank (National Centre for Biotechnology Information, NCBI) under the following accession numbers: MW078935 to MW078965 and MZ029056 to MZ029087, respectively for both genes (supplementary Table 1).

### MS spectra analysis

**Figure 3:**
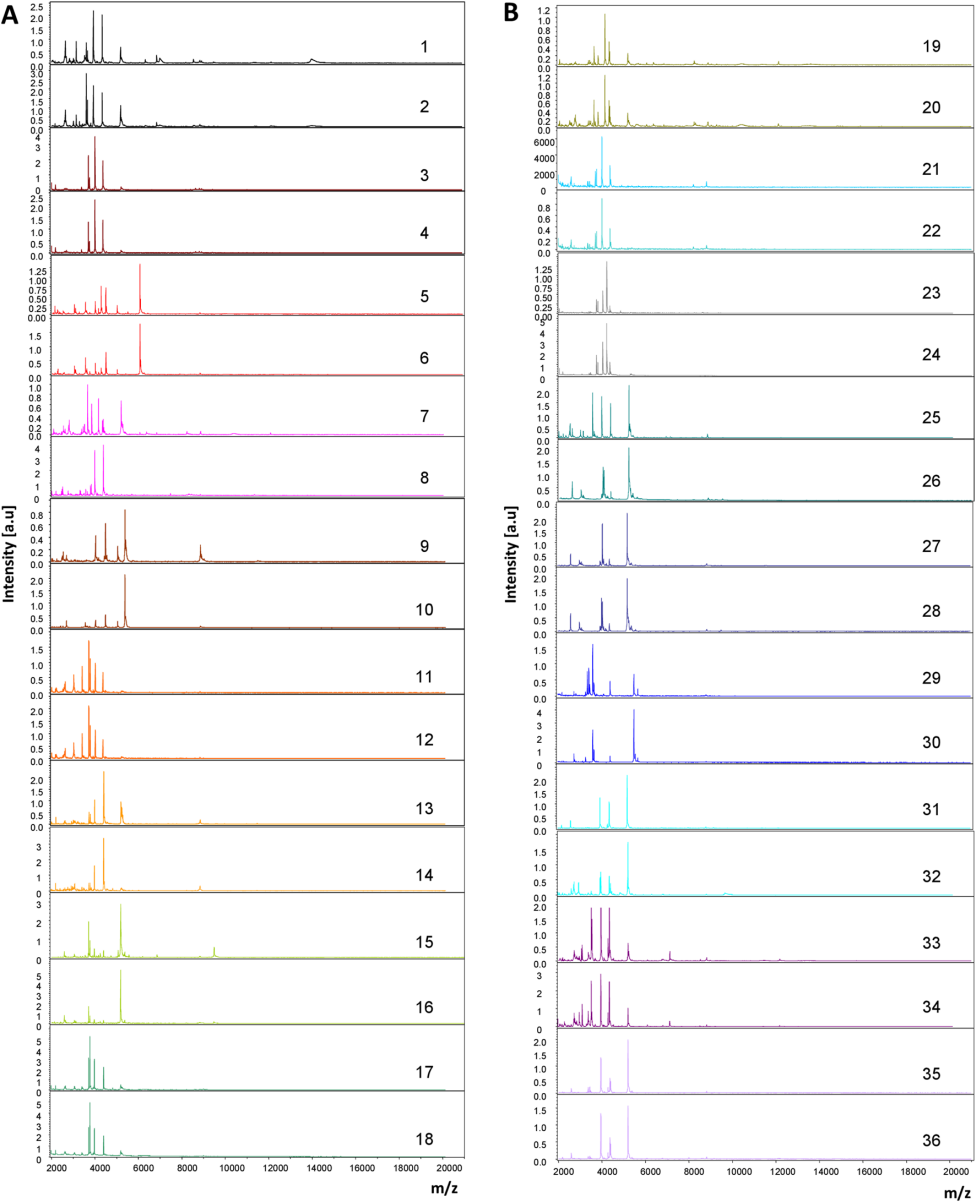
Representative MS profiles obtained for each termite species using the Flex analysis 3.3 software (**A**) and (**B**). (**A**) represents the spectra obtained from the leg protein of *Ancistrotermes cavithorax* (1–2), *Amitermes evuncifer* (3–4), *Cubitermes orthognatus* (5–6), *Cryptotermes brevis* (7–8), *Kalotermes flavicollis* (9–10), *Macrotermes bellicosus* (11–12), *Macrotermes herus* (13–14)*, Macrotermes ivorensis* (15–16), *Macrotermes subhyalinus* (17–18) and (**B**) the spectra from *Microcerotermes parvus* (19–20), *Microtermes sp* (21–22), *Odontotermes latericius* (23–24), *Procubitermes sjostedti* (25–26), *Promirotermes holmgreni* (27–28), (*Reticulitermes lucifugus* (29–30), *Trinervitermes geminatus* (31–32), *Trinervitermes occidentalis* (33–34), *Trinervitermes trinervius* (35–36). a.u.: arbitrary units; m/z: mass-to-charge ratio.

**Figure 4:**
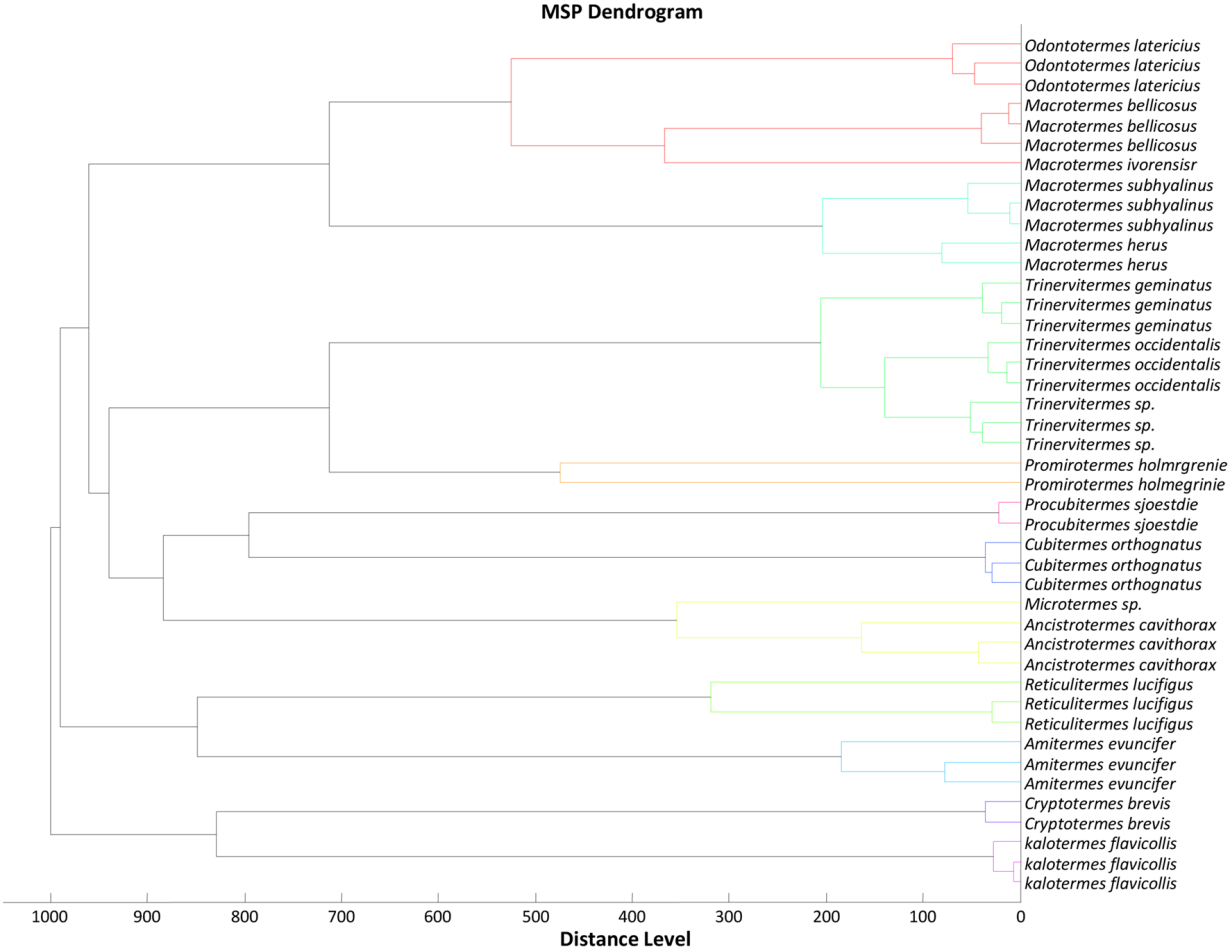
Dendrogram using 2 to 3 representative MS spectra from 16 distinct species treated with Maldi Biotyper v 3.0 software and the distance units correspond to the relative similarity of MS spectra.

In total, the legs of 432 termite specimens were subjected to MALDI-TOF/MS analysis. The visualization of the MS spectra obtained from all samples showed that they were of high quality (no smoothing, baseline subtraction corrects and peak intensity > 3000 arbitrary units,) (Fig. 3). A cluster analysis (dendrogram) was performed with two to five MS spectra from the legs of each species to evaluate the reproducibility and specificity of the spectra according to species. A perfect clustering of specimens of the same species on the same branch reveals intra-species reproducibility and inter-species specificity for the different termite species (Fig. 4). Interestingly, the MS spectra of sterile and reproductive caste specimens of the same species were specific according to caste as shown in the PCA and dendrogram made with two castes (reproductive and sterile) of *K. flavicollis* and *R. lucifugus* (Supplementary Fig. 2).

All the reference MS spectra included in our lab database have been deposited in a public repository in order to be shared with the entire research community, they are available and can be downloaded with the following DOI number: https://doi.org/10.35088/q281-st29.

### Blind test for validation of termite identification

The accuracy of MALDI-TOF MS identification of 17 termite species was evaluated by querying 389 specimens morphologically, identified against our updated MALDI-TOF MS database with 43 spectra of one to ten spectra per species confirmed by molecular biology (Table 2). However, the termite identified as *Microtermes* sp. was not included in the blind tested because there was only one specimen.

**Table 2:** Termite collection information (location, year of collection, morphological identification) and MALDI-TOF identification with log score value obtained by blind test.

Morphological identification	Collection country	Collection years	Number tested	Number added DB	MALDI-TOF MS identification (%)	Score rang LSV
*Ancistrotermes cavithorax*	Senegal	2019	4	1	*A. cavithorax* (100%)	[2.093–2.257]
*Amitermes evuncifer*	Senegal	2019	5	1	*A. evuncifer* (100%)	[1.993–2.421]
*Cubitermes orthognathus (Nitiditermes)*	Senegal	2019	10	2	*C. orthognathus (Nitiditermes)* (100%)	[1.705–2.253]
*Cryptotermes brevis*	Switzerland	2021	3	1	*C. brevis* (100%)	[2.041–2.350]
*Kalotermes flavicollis*	France	2013	10	2	*K. flavicollis* (100%)	[1.8–2.46]
*Macrotermes bellicosus*	Senegal	2019	25	3	*M. bellicosus* (100%)	[1.818–2.577]
*Macrotermes herus*	Mali	2016	3	1	*M. herus* (100%)	[2.780–2.322]
*Macrotermes ivorensis*	Togo	2010	2	1	*M. ivorensis* (100%)	[2.226–2.024]
*Macrotermes subhyalinus*	Senegal	2019	11	2	*M. subhyalinus* (100%)	[1.795–2.572]
*Microcerotermes parvus*	Senegal	2019	7	1	*Mi. parvus* (100%)	[2.531–2.853]
*Microtermes sp.*	Senegal	2019	0	1	–	–
*Odontotermes latericius*	Mali	2019	71	4	*O. latericius* (100%)	[2.001–2.581]
*Procubitermes sjostedti*	Senegal	2019	2	1	*Procubitermes sjostedti* (100%)	[2.448–2.455]
*Promirotermes holmgreni*	Senegal	2019	6	1	*P. holmgreni* (100%)	[2.492–2.599]
*Reticulitermes lucifugus*	France	2014–2021	113	11	*R. lucifugus* (100%)	[2.002–2.851]
*Trinervitermes geminatus*	Côte d'Ivoir/Senegal	2016/2019	56	4	*T. geminatus* (100%)	[1.924–2.403]
*Trinervitermes occidentalis*	Senegal/Cote d’Ivoir	2019	50	4	*T. occidentalis* (100%)	[1.93–2577]
*Trinervitermes trinervius*	Cote d'Ivoire	2016	11	2	*Trinervitermes* sp. *(100%)*	[1.921–2.342]

The result of the interrogation showed that 100% (389) of the species were correctly identified; i.e., they agreed with our morphological identification, with an LSV ranging from 1.65 to 2.851, mean of 2.290 ± 0.225, median of 2.299 (Table 2) and 98.4% (382) had LSVs > 1.8 (Supplementary Fig. 3). Looking at the level of each species for *A. cavithorax,* LSV identification ranged from 1.98 to 2.752, with a mean of 2.325 ± 0.236; for *A. evincifer* the LSVs ranged from 1.919 to 2.744 (2.2 ± 0.306); for *C. orthognathus,* from 1.938 to 2.813 (2.508 ± 0.271); for *C. brevis,* from 2.041 to 2.350 (2.181 ± 0.112); for *K. flavicollis,* from 1.729 to 2.547 (2.265 ± 0.231); for *M. bellicosus,* from 2.062 to 2.737 (2.459 ± 0.115); for *M. herus,* from 2.585 to 2.78 (2.678 ± 0. 068); for *M. ivorensis,* from 2.173 to 2.471 (2.322 ± 0.149) and for *M. subhyalinus* it ranged from 1.933 to 2.572 (2.309 ± 0.195). Similarly, *Mi. parvus* was identified, with LSVs ranging from 1.818 to 2.849 and a mean of 2.393 ± 0.225; *O. latericius,* with 1.963 to 2.581 (2.204 ± 0.111); *P. holmgreni,* with 1.719 to 2.846 (2.277 ± 0.257); *R. lucifugus,* with 1. 797 to 2.851 (2.329 ± 0.156); *T. geminatus,* with 1.65 to 2.693 (2.259 ± 0.165); *T. occidentalis,* with 1.727 to 2.642 (2.226 ± 0.171); *Trinervitermes* sp., with 1.817 to 2.515 (2.066 ± 0.200) and one *P. sjostedti* identified with 2.806.

## Discussion

According to our morphological identification, 22 species belonging to 12 genera were identified in this study. The presence of most of the species identified in West Africa had already been reported in this area^26^. Termites belonged to the reproductive or biological caste (with wings) and sterile caste composed of workers and soldiers (without wings). Each caste ensures a specific role within the colony; thus, the soldiers are involved in defense, workers in elementary tasks (gathering food, taking care of the queen and king and constructing or repairing the nest) and the reproductive caste ensure the function of reproduction^27^.

The molecular biology of social insects has always been contentious^28^; we used both gene systems including *12SrRNA* and *COI* in our study. Based on previous studies the *12SrRNA* gene was suggested as more discriminating compared to the *COI* gene^29,30^. However, molecular identification of Isoptera has most often used the *COI* gene^29,31,32^. Additionally, *12S* is characterized by the narrow spectrum of sequences for each species where each species is presented by one or two sequence.

Based on the results of molecular biology, our morphological identification was confirmed for some species, such as *K. flavicollis*, *M. subhyalinus*, *R. lucifugus*, *T. geminatus*, *T. occidentalis*, *C. brevis*, *A. evuncifer, A. cavithorax*, *M. bellicosus*, *M. subhyalinus*, *M. herus* and *O. latericius* by both genes or only by the *COI* gene, whose homologous sequences were available on Genbank. However, for ten species (*M. ivorensis*, *Mi. parvus*, *P. sjostedti*, *R. santonensis*, *R. grassei, P. holmgreni*, *T. trinervoides*, *T. togoensis*, *T. trinervius*) morphological identification did not match with molecular identification. Most probable explication is the morphological misidentifications that we made due to close anatomical similarities among species from *Reticulitermes* and *Trinervitermes* genera^33,34^. On the other hand, this discrepancy between our morphological and molecular identification would be due to the updating of the systematics of the termites with a change in the name of the genus as in the case of the genus *Cubitermes* changed to *Nitiditermes*^35,36^ or by the lack of reference sequence of some species in the GenBank database, as in the case of the species *M. ivorensis* and *P. holmgreni*, which is one of the limitations of the molecular method^21^.

In this study we developed the MALDI-TOF MS tool to identify different termite species. Although some studies have been performed on the identification of the gut microbiota and chemical composition of termites using MALDI-TOF MS^26,37,38^, to our knowledge our study is the first to use this tool for the identification of these insects. MALDI-TOF MS is a technique that allows the identification of bimolecular protein contained in a sample by soft ionization according to the mass/charge ratio (m/z)^21^. The measurement of the m/z ratio is determined by the time it takes for an ion to travel the flight path and thus generate a spectral profile specific to the composition of the sample being analyzed. Over several years, this technique has been developed for the routine diagnosis of a large number of microorganisms (bacteria, archaea, yeasts, filamentous fungi, helminths and intestinal protozoa) of medical and/or veterinary importance^21^. Over the last 15 years, MALDI-TOF MS has been widely used in entomology for the identification of a large number of arthropod vectors and non-vectors, as well as for the determination of blood meal origin and the discrimination of infected and non-infected arthropods. Although this technique is less expensive, reliable, fast and does not require knowledge of entomology, its use in entomology requires the development of specific protocols to generate reproducible and species-specific spectra^21^. The price of the machine, the choice of the part of the arthropod used for MALDI-TOF MS analysis and the method of preservation of the arthropods, are limiting elements of this technique^21^. In our study, 100% of the species were correctly identified; i.e., they agreed with our morphological identification, with an LSV ranging from 1.705 to 2.862 (mean: 2.302 ± 0.254) and 97% of specimens had LSVs > 1.8. The high rate of correct identification, proving the performance of the MALDI-TOF MS tool in distinguishing the different arthropod species, has already been reported in several studies^21^. It is interesting to note that very closely related species, often difficult to distinguish morphologically, (species of the genus *Macrotermes* and *Trinervitermes*) and specimens of different castes (reproductive and sterile), could be distinguished by MALDI-TOF MS. The ability of MALDI-TOF MS to distinguish closely related tick species has already been reported^39^.

To summarize, our study reliably shows that MALDI-TOF/MS is a promising tool that may make termite studies much easier and specific. It would be interesting to apply this innovative tool on termites from other sites in the world in order to have a maximum of species to enrich our MALDI-TOF MS database.

## Materials and methods

### Study area and collection periods

Termites were collected in two European countries (France and Switzerland) and in four West African countries: (Mali, Ivory Coast, Togo and Senegal). The termites from France were collected in a heavily infested house in an old building located in Châteauneuf-Les-Martigues near Marseille. The pictures taken show the damage caused by the termites. The termites from Marseille were sampled in 2013, 2014 and 2021, those from Togo in 2010, Mali and Ivory Coast in 2016, Senegal in 2019 and Switzerland in 2021.

The collection in Senegal was made at four sites; Niokolo-Koba National Park (sites of Simenti, Dar Salam and Niokolo Poste) and in Southeastern Senegal (region of Kédougou) in the Dindefello forest, were the termitaries were identified visually, and samples collected using a shovel and pincer. From each termitary, the soil substrate, the fungus combs (if available) and adult termites (soldiers and workers) were collected in a plastic ventilated box where they were stored during the transport at ambient temperature. On arrival, termites were separated from the substrate and fungus. The maps showing the collection sites were made with QGIS software version 3.20 (Fig. 1). All termites were stored in 70% ethanol except for termites collected from Marseille in 2020. They were stored at − 20 °C because they were collected in the proximity of the laboratory.

### Morphological identification

Morphological identification of termites was done down to the genus and/or species level using different keys based on the specific and discriminating characteristics of soldiers, workers and alates, or biological, such as the keys of Sands^40^, Clay^41^, Adlard et al.^42^, Ifan^43^, Bouillon^44^.

The termite collection site was also an important element that was considered and helped us in the taxonomy.

For workers of some species, we dissected the mandible to identify termites down to the species. The criteria were observed with the optical microscope, the binocular loupe, and the ZEISS Axio Zoom V16, and then photographed with the digital Canon E05 7D supplied with a Canon MP-E 65 mm Lens (French).

### Molecular identification of termites

To confirm the morphological identification of termites whose spectra were to be introduced into our MS spectra database (DB), molecular analyses were performed. DNA was extracted from a small part of the termite abdomen, which was subjected to enzymatic lysis by incubation at 56 °C overnight in 180 μL of lysis buffer G2 (QIAGEN, Hilden, Germany) and 20 μl of proteinase K (QIAGEN, Hilden, Germany). Total DNA was extracted into 100 μL of eluate using EZ1 Tissue Kit (Qiagen, Hilden, Germany) according to the manufacturer's instructions and stored at − 20 °C before use.

Molecular identification of termites at the species level was performed by sequencing the standard PCR product of a fragment of the small subunit ribosomal RNA (*12S rRNA*) gene (12S-F SR-J-141995: 5′TACTATGTTACGACTTAT-3′/12S-R SR-N-14594: 5′AAACTAGGATTAGATACCC-3′) and the cytochrome c oxidase I gene *(COI)* (LCO1490: 5′-GGTCAACAAATCATAAAGAYATYGG-3′ and dgHCO2198: 5′-TAAACTTCAGGGTGACCAAARAAYCA-3′) as previously described^29,45^. PCR products were separated and visualized by electrophoresis in 1.5% agarose. All positive samples were purified and directly sequenced using the commercial Big Dye Terminator Cycle Sequencing Kit (Perkin Elmer Applied Biosystems, Foster City, CA, USA) with an ABI automated sequencer (Applied Biosystems). Obtained sequences were edited using ChromasPro software (ChromasPro 1.7, Technelysium Pty Ltd., Tewantin, Australia) and compared for similarity to sequences available in the GenBank (https://blast.ncbi.nlm.nih.gov/Blast.cgi). Molecular phylogenetic and evolutionary analyses were conducted in TOPALi2.5 (http://www.topali.org/).

### Termite preparation for MALDI-TOF analysis

After rinsing and drying on sterile filter paper, three legs from each of the termites were individually placed in an Eppendorf tube and dried at 37 °C overnight for those stored in alcohol. Specimens that were at − 20 °C were not dried at 37 °C overnight. The legs were then homogenized with the TissueLyser (Qiagen) with a pinch of glass beads and 20 µL of a mixture of 70% formic acid and 50% acetonitrile (Fluka, Buchs, Switzerland) in a three-minute cycle at a frequency of 30 Hertz as already described^20,46,47^. The legs of *Aedes albopictus* reared in our laboratory were used as a positive control in all manipulations.

### MALDI-TOF/MS parameters

Protein mass profiles were obtained using a Microflex LT MALDI-TOF Mass Spectrometer (Bruker Daltonics, Germany), with detection in the linear positive-ion mode at a laser frequency of 50 Hz within a mass range of 2–20 kDa. The setting parameters of the MALDI-TOF/MS apparatus were identical to those previously used^20,48,49^. Briefly, the acceleration voltage was 20 kV, and the extraction delay time was 200 ns. Each spectrum corresponds to ions obtained from 240 laser shots performed in six regions of the same spot and automatically acquired using the AutoXecute of the Flex Control v.2.4 software (Bruker Daltonics).

### Spectra analysis

The MS spectra were then exported to flex Analysis v3.3, ClinProTools v2.2 and MALDI-Biotyper v3.0. (Bruker Daltonics) software for data processing (smoothing, baseline subtraction, peak picking). The quality of MS spectra was evaluated by visualization of spectra obtained from the four spots for each sample with the flex Analysis v3.3 software (Bruker Daltonics). Cluster analyses (MSP dendrogram) and principal component analysis (PCA) were performed to verify intra-species reproducibility and inter-species specificity as well as variability within different castes (soldiers and workers) of the same species. Cluster analyses were performed based on the comparison of the MSPs given by the MALDI-Biotyper v3.0. software and grouped according to the mass profile of the proteins (i.e., their mass signals and intensities) and it reflects how tick specimens are related to each other. The setting parameters were as follows: distance measure by correlation, linkage by average; the score threshold value for a single organism was 300 (arbitrary unit) and for related organisms was 0 (arbitrary unit).

### Database creation and blind test for validation of termite identification

Reference MS spectra were created from the spectra of each termite species, where available, using MALDI-Biotyper v3.0. (Bruker Daltonics). MS spectra of legs from 42 specimens of termites identified morphologically and molecularly were added into our MS spectra database (DB)^50^, *Microtermes* sp. spectra were not added to the DB because we had only one specimen.

All remaining spectra were blind tested against our MS spectra database for termite identification. The level of the identification significance was established using the log score values (LSVs) given by the MALDI-Biotyper v.3.3 software that correlated with a corresponding degree of signal intensity of the request and reference mass spectra. The LSVs, ranging from 0 to 3, were obtained for each spectrum of the tested samples. The results of identification were considered reliable and relevant when the LSVs were greater than or equal to 1.8, as previously established in many studies^20,49^.

## Supplementary Information

Supplementary Figure 1.

Supplementary Figure 2.

Supplementary Figure 3.

Supplementary Table 1.

## Data Availability

All relevant data are within the manuscript and its Supporting Information files.
