# Supplementary material for: Use of the proteomic tool MALDI-TOF MS in termite identification
Source: Sci Rep. 2022 Jan 14;12:718. doi: 10.1038/s41598-021-04574-0 (PMC8760289; doi:10.1038/s41598-021-04574-0)
Supplement: Supplementary file 1 — Supplementary Figure 1. [file 41598_2021_4574_MOESM1_ESM.pdf]

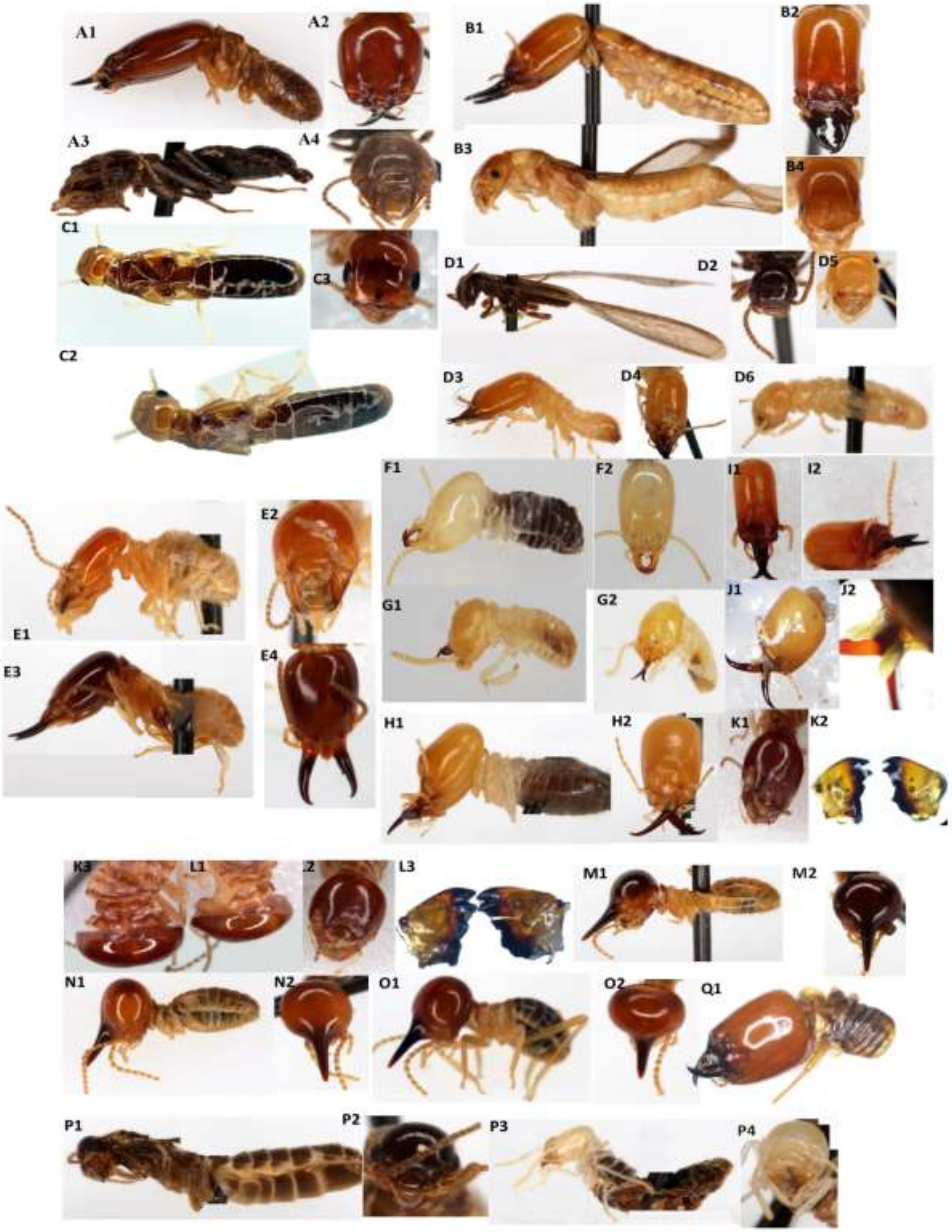

Photographs of identified termite species and morphological characteristics taken with a Zeiss Axio Zoom V16 stereomicroscope (Zeiss, Marly le Roi, France) and the digital Canon E05 7D supplied with a Canon MP-E 65 um Lens (French)
