# Supplementary material for: Use of the proteomic tool MALDI-TOF MS in termite identification
Source: Sci Rep. 2022 Jan 14;12:718. doi: 10.1038/s41598-021-04574-0 (PMC8760289; doi:10.1038/s41598-021-04574-0)
Supplement: Supplementary file 2 — Supplementary Figure 2. [file 41598_2021_4574_MOESM2_ESM.pdf]

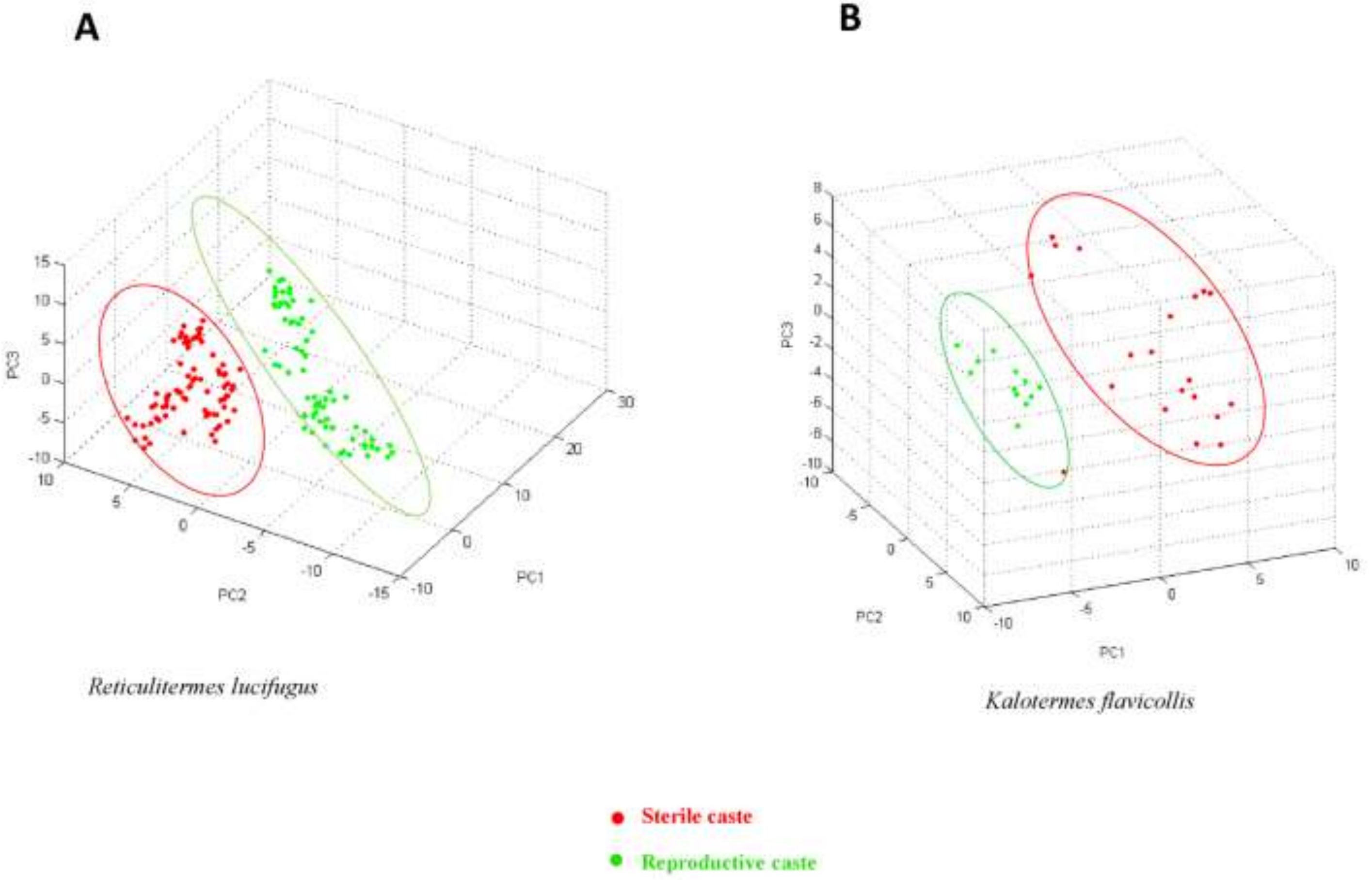

MALDI-TOF MS distinction of spectra of the reproductive caste (green) and sterile caste (read) **(A)** *Reticulitermes lucifugus* and **(B)** *Kalotermes flavicollis* illustrated on principal component analysis using ClinProTools 2.2 software. a.u.: arbitrary units; m/z: mass-to-charge ratio
