# Supplementary material for: Use of the proteomic tool MALDI-TOF MS in termite identification
Source: Sci Rep. 2022 Jan 14;12:718. doi: 10.1038/s41598-021-04574-0 (PMC8760289; doi:10.1038/s41598-021-04574-0)
Supplement: Supplementary file 3 — Supplementary Figure 3. [file 41598_2021_4574_MOESM3_ESM.pdf]

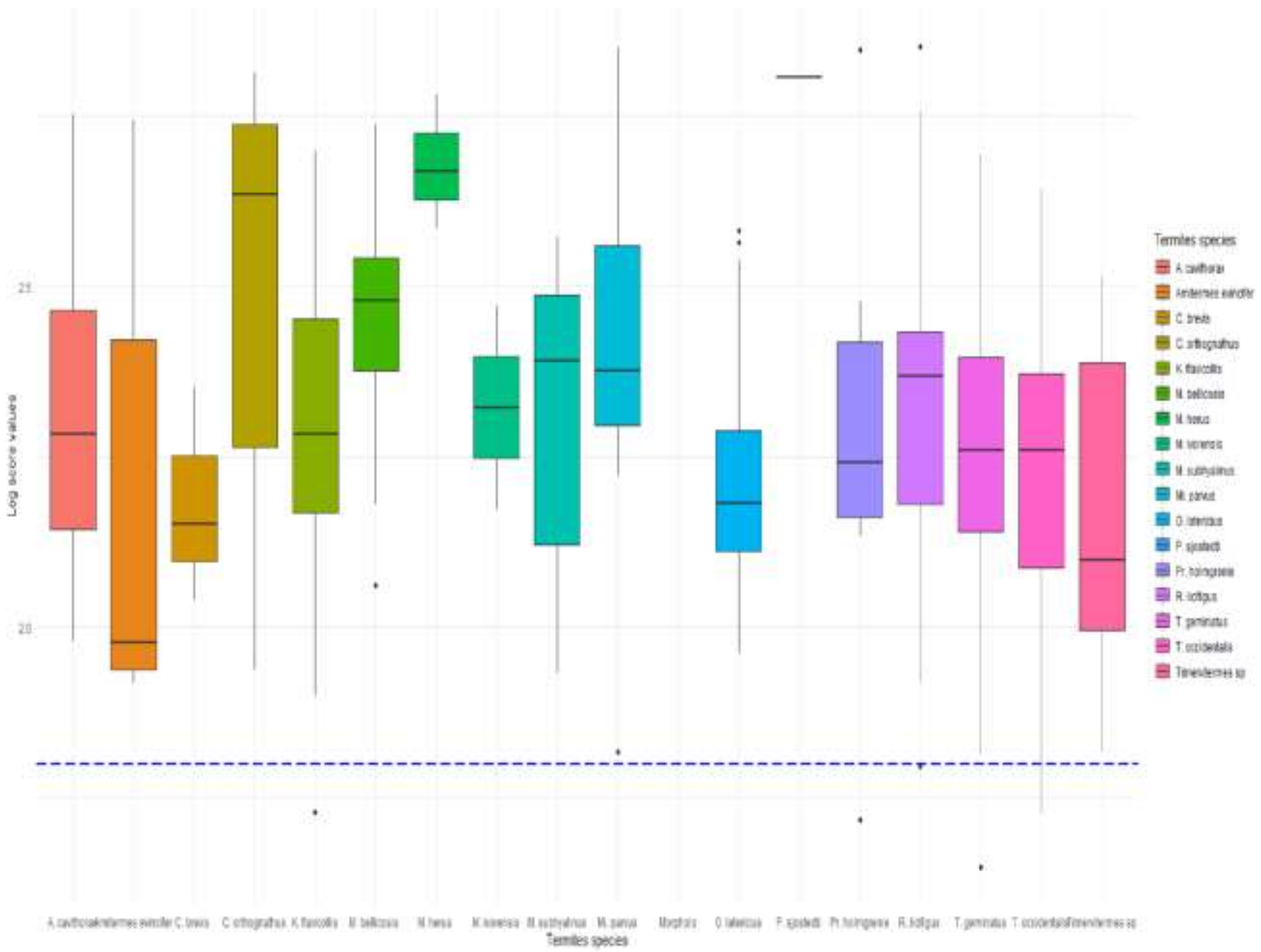

Box plot showing the distribution of log score values obtained by blind testing of different termite species. The blue line represents the limit of the correct identification threshold of 1.8
