# Supplementary material for: Use of the proteomic tool MALDI-TOF MS in termite identification
Source: Sci Rep. 2022 Jan 14;12:718. doi: 10.1038/s41598-021-04574-0 (PMC8760289; doi:10.1038/s41598-021-04574-0)
Supplement: Supplementary file 4 — Supplementary Table 1. [file 41598_2021_4574_MOESM4_ESM.docx]

**Supplementary Table 1:** list of different termite species and the GenBank accession numbers for the *COI* and *12s rRNA* genes

| Termite species | Accession number of *COI* gene | Accession number of *12SrRNA* gene |
| --- | --- | --- |
| *Macrotermes bellicosus* | MZ029063- MZ029065 | MW078940- MW078941 |
| *Macrotermes subhyalinis* | MZ029066- MZ029067 | MW078944- MW078945 |
| *Macrotermes herus* | MZ029068 | MW078952 |
| *Macrotermes ivorensis* | / | MW078950 |
| *Trinervitermes geminatus* | MZ029079- MZ029081 | MW078956 |
| *Trinervitermes occidentalis* | MZ029082- MZ029083 | MW078954- MW078955 |
| *Trinervitermes sp* | MZ029084- MZ029085 | MW078965 |
| *Trinervitermes trinervius* | MZ029086-MZ029089 | MW078961- MW078964 |
| *Odontotermes latericius* | MZ029069-MZ029072 | MW078935-MW078938 |
| *Reticulitermes lucifugus* | MZ029075- MZ029078 | MW078939 |
| *Kalotermes flavicollis* | MZ029061- MZ029062 | MW078942- MW078943 |
| *Microcerotermes parvus* | / | MW078957- MW078958 |
| *Promirotermes holmgreni* | MZ029074 | MW078946- MW078947 |
| *Nitiditermes prolaxius* | MZ029073 | MW078951 |
| *Nitiditermes sp* | MZ029059- MZ029060 | MW078959- MW078960 |
| *Amitermes evincifer* | MZ029057- MZ029058 | MW078948 |
| *Ancistrotermes cavithorax* | MZ029056 | MW078949 |
